# Platypnea-Orthodeoxia Syndrome: Manifestation of a Patent Foramen Ovale

**DOI:** 10.7759/cureus.75970

**Published:** 2024-12-18

**Authors:** João Oliveira, Martina Arandjelovic, Inês Pintor, Daniela Meireles, Manuela Vieira, Tiago Adrega, Joana Neves

**Affiliations:** 1 Internal Medicine, Hospital Infante D. Pedro, Aveiro, PRT; 2 Infectious Diseases, Hospital Infante D. Pedro, Aveiro, PRT; 3 Cardiology, Hospital Infante D. Pedro, Aveiro, PRT

**Keywords:** acute ischaemic stroke, hypoxaemia, interatrial septal aneurysm, patent foramen ovale (pfo), platypnea-orthodeoxia syndrome

## Abstract

Platypnea-orthodeoxia syndrome (POS) is a rare clinical condition characterized by dyspnea and hypoxemia during orthostatism, with relief in the supine position. The diagnosis of POS requires a high clinical suspicion, and its etiology stems from the admixture of venous blood, poor in oxygen, with arterial blood via a shunt. A patent foramen ovale (PFO) is the most commonly encountered anomaly at the root of POS.

Here, we present the case of a 67-year-old female patient where suspected POS led to the diagnosis of a PFO, which was the cause of an ischemic stroke.

## Introduction

First described in 1949 by Burchell et al., platypnea-orthodeoxia syndrome (POS) is a rare clinical condition characterized by dyspnea and hypoxemia in the orthostatic position, platypnea, and orthodeoxia, respectively [1-3]. The exact prevalence is unknown, but over 200 cases have been reported since it was first described [4].

Although there is no consensus regarding the definition, POS is considered when arterial oxygen pressure falls by > 4 mmHg or arterial oxygen saturation drops by > 5% when switching from the supine to orthostatic position [1,2]. Typically, the severity of hypoxemia is mild; however, some patients experience a significant reduction in arterial oxygen pressure [4].

The pathophysiological mechanism of POS is explained by the admixture of deoxygenated venous blood with oxygenated arterial blood via a shunt. Depending on the location of the shunt, POS can be classified as intracardiac, extracardiac (intrapulmonary shunt or ventilation/perfusion mismatch), or attributed to various other phenomena [1,4,5]. Intracardiac POS requires right-to-left communication between the heart chambers, which most commonly occurs at the interatrial septum, where anomalies such as a patent foramen ovale (PFO), interatrial septal defect (ASD), or interatrial septal aneurysm (ASA) may be present. A PFO is the most common cause of an intracardiac shunt and was the cause of the POS (associated with anatomical changes such as atrial septal defects and atrial septal aneurysms) detected in the clinical case presented here [5,6].

## Case presentation

The authors present the case of a 67-year-old female patient with a medical history of hypertension and dyslipidemia receiving optimized medical treatment; non-insulin-dependent type 2 diabetes mellitus; obesity class 2; hypothyroidism medicated with levothyroxine 50 mcg; and a cryptogenic ischemic stroke (left total anterior circulation stroke) in April 2024 for which the patient underwent primary thrombectomy and maintained mild dysarthria as a sequela. In September 2024, she was sent to the emergency department due to an altered level of consciousness that was characterized by temporal and spatial disorientation along with psychomotor agitation. Upon arrival, she was hemodynamically stable, apyretic, and had a peripheral oxygen saturation of 90% on ambient air. Upon objective examination, she appeared drowsy, with colored and hydrated skin and mucous membranes; eupneic; her cardiac auscultation was regular, with no audible murmurs; pulmonary auscultation was unremarkable; and she presented no peripheral edema. Arterial blood gas identified hypoxemic respiratory failure (pH 7.40; pCO2 35.0 mmHg; pO2 59.1 mmHg; HCO3- 22.2 mEq/L), while routine blood work and urinalysis were within normal limits. Immunochromatography testing was negative for SARS-CoV-2, respiratory syncytial virus, adenovirus, and influenza A and B. Her chest X-ray showed no acute pleuroparenchymal changes; the electrocardiogram identified sinus rhythm with a normal heart rate; and the cranioencephalic computed tomography scan revealed an ischemic cortical-subcortical lesion in the left temporoparietal and frontal regions from her prior ischemic stroke.

**Figure 1:**
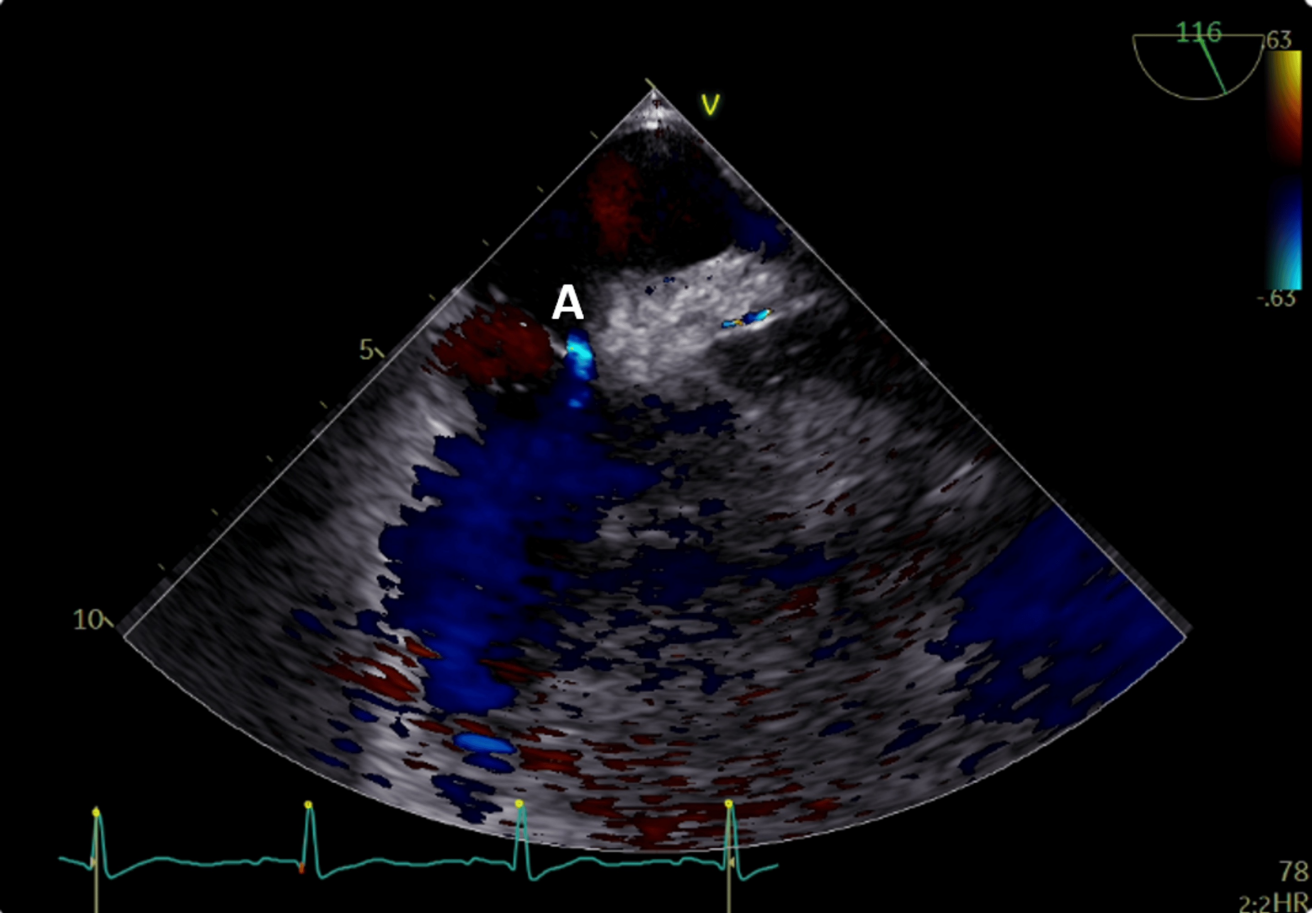
Transesophageal echocardiogram with color Doppler showing the patent foramen ovale (A).

**Figure 2:**
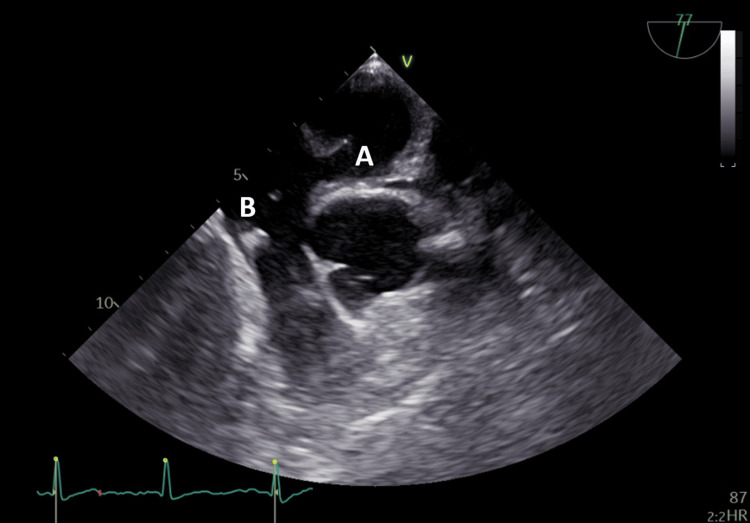
Transesophageal echocardiogram showing patent foramen ovale (A) and Eustachian valve (B).

**Figure 3:**
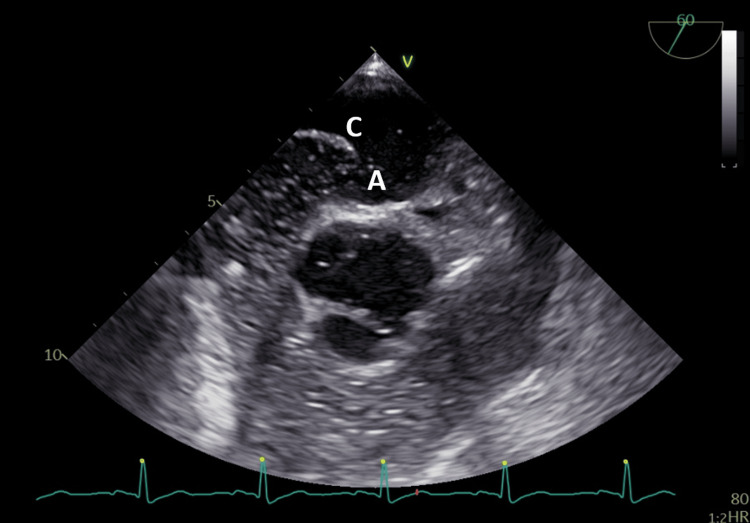
Transesophageal echocardiogram with bubble test showing patent foramen ovale with shunt (A) and atrial septal aneurysm (C).

**Figure 4:**
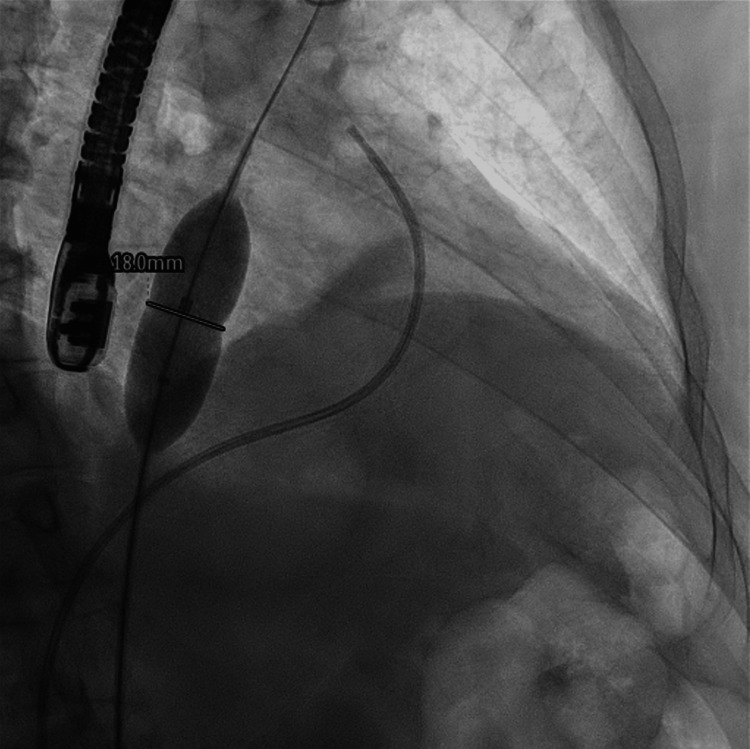
Fluoroscopy image showing the diameter of the patent foramen ovale.

**Figure 5:**
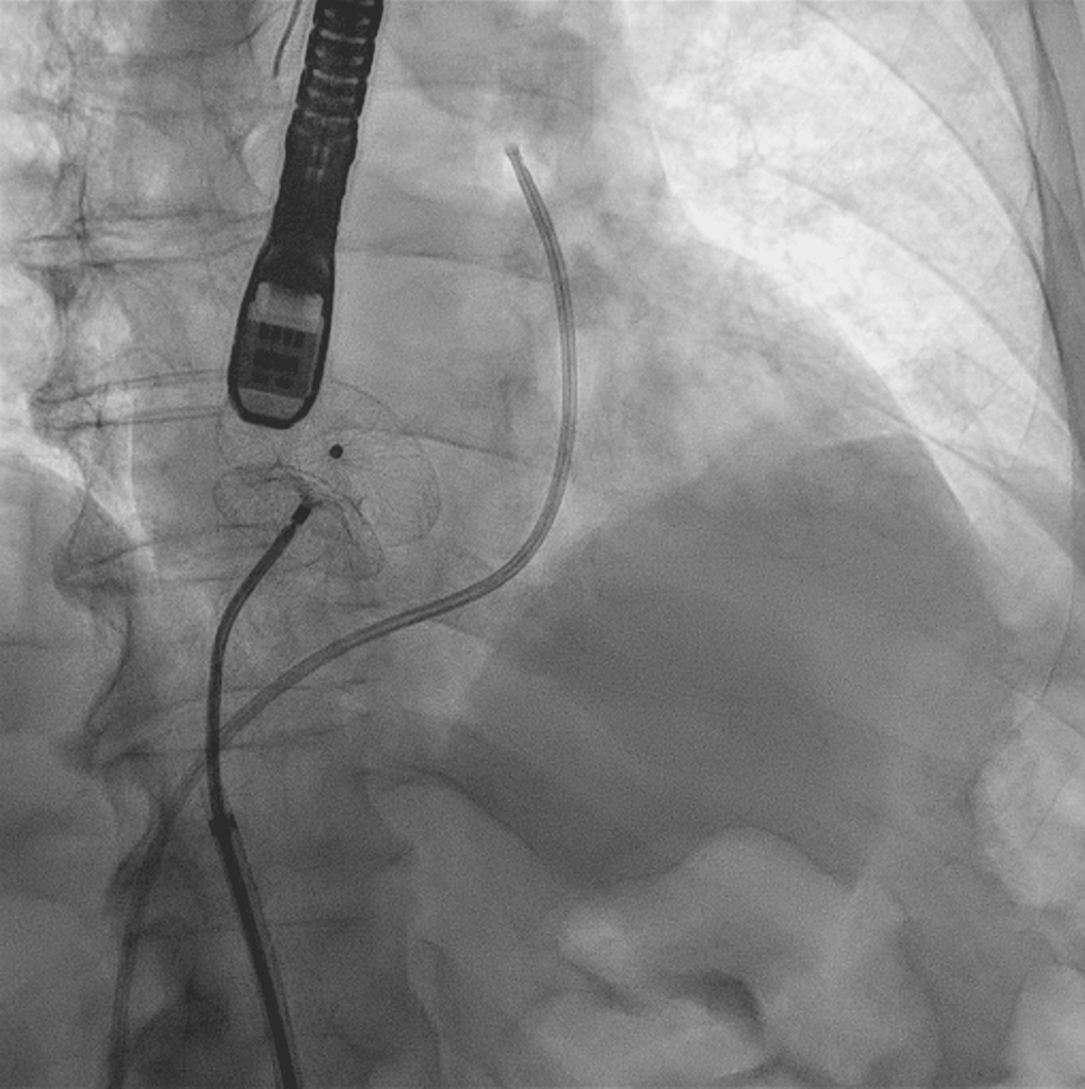
Fluoroscopy image showing the Amplatzer ASD device (Abbott Laboratories, Chicago) implanted.

The patient was admitted to an internal medicine ward for a diagnostic investigation of her altered level of consciousness and hypoxemic respiratory failure. During this hospitalization, the results of the diagnostic evaluation of the ischemic stroke she suffered in April of the same year were reviewed: the Holter monitor had recorded sinus rhythm with rare supraventricular and ventricular ectopic heartbeats; the transthoracic echocardiogram demonstrated good overall and segmental systolic function without significant valvular abnormalities; and the carotid Doppler ultrasound revealed no stenosis. During the hospital stay, the patient experienced frequent episodes of tachypnea accompanied by peripheral desaturation, with an increasing need for supplemental oxygen. Given the uncertain etiology of the stroke the patient suffered in April and her current hypoxemic respiratory failure, she underwent a more detailed assessment of cardiac function and morphology due to high clinical suspicion of interatrial communication. A transesophageal echocardiogram with a bubble test was performed, which confirmed findings consistent with a bidirectional atrial shunt, spontaneous right-to-left foramen ovale, Eustachian valve, and atrial septal aneurysm (Figures 1-3). Additionally, arterial blood gases confirmed platypnoea-orthodeoxia syndrome. With supplemental oxygen (12 L/min), the pO2 in the upright position was 85 mmHg, compared to 93 mmHg in the supine position, resulting in a differential of 8 mmHg. The case was discussed with the Cardiology Department and subsequently with Interventional Cardiology, which admitted the patient electively for percutaneous closure of the interatrial defect. During the procedure, a patent foramen ovale with a diameter of 18 mm and other findings were identified. Balloon sizing was performed, and a 20 mm Amplatzer ASD device (Abbott Laboratories, Chicago) was successfully implanted (Figures 4, 5). The patient became asymptomatic, and the hypoxemic respiratory failure resolved.

## Discussion

POS requires a high degree of clinical suspicion in patients with dyspnea; it is a rare condition, and its prevalence is likely underestimated [4,5].

The patient, in this case, had a PFO, which was probably the cause of her ischemic stroke in April 2024, and POS was diagnosed during hospitalization five months later. In the adult population, the prevalence of PFO is 20-25%, and in the case of patients who have suffered a cryptogenic stroke, the prevalence reaches 40-50%. An atrial septal aneurysm (ASA) associated with a PFO increases the risk of stroke due to thrombus formation in areas of blood flow stagnation; the added presence of an Eustachian valve can further contribute to this risk by directing blood flow from the inferior vena cava towards the PFO [7,8]. In the case of our patient, she exhibited both of these features in association with the PFO, which increased her risk of stroke [7].

Although it was not performed at the time of the patient's ischemic stroke, a transesophageal echocardiogram with bubble study was conducted during the second hospitalization since there was high clinical suspicion of a shunt. This type of echocardiography is considered the gold standard for diagnosing PFO and assessing its dimensions and anatomical features. Some centers opt for an initial screening using transcranial Doppler ultrasound (it also has the advantage of being a much less invasive exam). However, transcranial Doppler ultrasound is sensitive but less specific in differentiating between cardiac and pulmonary shunts [7-9].

In the specific context of cryptogenic stroke, the benefit of PFO closure depends on how likely it is to be the underlying cause compared with other factors, as determined by the Risk of Paradoxical Embolism (RoPE) score. Patients with a RoPE score above seven have a higher probability of their stroke being caused by a PFO and therefore would most likely benefit from percutaneous closure compared to medical treatment alone [8].

Definitive treatment of POS requires correcting the interatrial defect, typically through percutaneous or surgical closure of the PFO. Currently, the percutaneous approach supplants heart surgery due to its lower mortality rate and cost [2,4,10].

The Amplatzer device, implanted percutaneously and utilized in the case of our patient, is the most effective in reducing cryptogenic strokes since it achieves complete PFO occlusion in the majority of cases. It demonstrates superior clinical outcomes compared to alternatives and is rarely associated with thrombus formation or embolization. Percutaneous PFO closure improves dyspnea in 95% of patients with POS, and oxygen saturation also increases by 10-20% when standing [1,2,4,8,11,12].

## Conclusions

Although the patient had suffered an ischemic stroke previously in the year and during her second hospitalization, she presented clinical findings consistent with POS, recognizing the latter was crucial in arriving at the diagnosis of a PFO. A thorough etiological investigation is paramount when faced with a patient who suffered a cryptogenic stroke. Once large artery atherosclerosis, cardioembolism, and small vessel occlusion have been excluded, it is essential to consider a PFO as a potential cause.

Numerous patients presenting with dyspnea and hypoxemia are regularly hospitalized, and in some cases, diagnosis may not always be linear. POS is a particularly challenging entity in this respect; complex pathophysiology coupled with low prevalence has likely led to underdiagnosis. Therefore, this case serves as a reminder to consider POS in patients with similar semiology.

## References

[1] Agrawal A, Palkar A, Talwar A (2017). The multiple dimensions of platypnea-orthodeoxia syndrome: A review. Respir Med.

[2] Akin E, Krüger U, Braun P (2014). The platypnea-orthodeoxia syndrome. Eur Rev Med Pharmacol Sci.

[3] Knapper JT, Schultz J, Das G, Sperling LS (2014). Cardiac platypnea-orthodeoxia syndrome: an often unrecognized malady. Clin Cardiol.

[4] Salas-Pacheco JL (2022). Mechanisms of platypnea-orthodeoxia syndrome. Arch Cardiol Mex.

[5] Soares PR, Melo N, Ferrao D, Sousa E, Santos A, Gomes A, Friões F (2022). Platypnea-orthodeoxia syndrome: a rare cause of positional respiratory failure. Cureus.

[6] Santos F, Teixeira Reis A, Pessoa A, Agudo M, Brigas D (2023). Platypnea-orthodeoxia syndrome: two case reports. Cureus.

[7] Mojadidi MK, Zaman MO, Elgendy IY (2018). Cryptogenic stroke and patent foramen ovale. J Am Coll Cardiol.

[8] Gonnah AR, Bharadwaj MS, Nassar H, Abdelaziz HK, Roberts DH (2022). Patent foramen ovale: diagnostic evaluation and the role of device closure. Clin Med (Lond).

[9] Vitarelli A (2019). Patent foramen ovale: pivotal role of transesophageal echocardiography in the indications for closure, assessment of varying anatomies and post-procedure follow-up. Ultrasound Med Biol.

[10] Meier B (2014). Patent foramen ovale and closure technique with the amplatzer occluder. Scientifica (Cairo).

[11] Stortecky S, da Costa BR, Mattle HP (2015). Percutaneous closure of patent foramen ovale in patients with cryptogenic embolism: a network meta-analysis. Eur Heart J.

[12] Giblett JP, Williams LK, Kyranis S, Shapiro LM, Calvert PA (2020). Patent foramen ovale closure: state of the art. Interv Cardiol.

